# Gout

**Published:** 1918-11

**Authors:** N. D. Buie

**Affiliations:** Marlin, Texas


### ORIGINAL ARTICLES.

### Gout.

(Podagra, Arthritis Urica.)

N. D. BUIE, M. D., MARLIN, TEXAS.

Definition.—Gout is a constitutional disease caused by an excess of uric acid and other purin bases in the blood, manifesting itself by attacking various tissues of the body, especially the articulations. The disease is always accompanied by kidney change which renders the kidneys less permeable to uric acid and causes the accumulation of this substance in the blood. There is present a *uric acid* diathesis, which pre-disposes to faulty metabolism and incomplete oxidation of the purin bases to soluable urea.

The disease has been a classic for medical writers since the days of Hippocrates, who first described it. It, however, has only recently, had its clearer differentiation from other joint manifestations since our exhaustive studies of focal infections, which studies have left gout as one of the very few diseases which we would classify still as metabolic and not microbic in origin. Yet, since we have not been able to determine whether this disease, accompanied as it always is by a distinct kidney pathology, is the cause or result of a certain nephrosis, it is possible that in the near future gout may cease to be a disease entity and become recognized as only a symptom.

### ETIOLOGY.

Gout is more common between the ages of 30 to 50, and much more common in males. It may occur in children, but seldom begins after 50 years of age. It has a predilection for the followers of Bacchus and gormandizers of Epicurean talents. Gout selects its victims from the idle rich and the gluttons of the "Sons of Rest". Brain workers with sedentary habits furnish a fertile soil.

The laborer with his ale, porter, poor food, and exposure to cold, gives to us the type called "poor man's gout".

The above causes are incidental. The real cause is the faulty metabolism of purins or nucleoprotiens with inadequate kidney function for the elimination of the end products of these bodies, which cause their accumulation in the blood stream. The nucleoproteins cannot be oxidized here past the uric acid stage. And the chlorides under certain conditions act on the uric acid causing the formation of quadriurate of soda which is further broken down into biurate of soda, and this is deposited about the joints causing resultant tophi and symptoms.

Why does gout have such a predilection for the metatarsophalangeal articulation, and why do attacks so often come during the early morning hours following "a night before"? When we remember the factors which produce an attack of gout, viz.: excess of uric acid producing food, lowered vitality from the debauch, excess of sodium chloride, slowing of the circulation and lowering the body temperature, we know these factors will bring about a precipitation of the biurate of soda. When we remember that the sodium chloride content of the blood is 7 per cent., of synovia 8 per cent., and of cartilage 9 per cent. we can see why joints are attacked first. The debauchee arrives home at 2 a. m. and retires with feet cold. Wine has lowered his temperature and circulation. The great toe joint is the fartherest large joint from the heart, and it is here the uric acid meets with an abundant supply of sodium chloride which makes the conditions for the formation of the pernicious sodium biurate ideal, and the precipitation takes place. By 3 a. m. the big toe joint has doubled its size, and the erstwhile gallant of the evening before disturbs the peaceful slumbers of his neighbors.

SYMPTOMS.

In the acute stage the symptoms come on suddenly, usually at night following indiscretions in diet and drink. The patient is awakened by a sharp cutting pain in a distant large joint, most often that of the big toe. The joint rapidly swells, becoming red and glazed. There is great tenderness elicited by touch or attempted movement. There is often nausea and vomiting with rise of temperature to 102 to 103, dizziness, costive bowels and the urine which had previously been scanty becomes more abundant, and is loaded with urates, some chlorides, a few hyaline and granular casts and a trace of albumen. The face is flushed and the pulse is full and bounding. Frequent acute attacks incompletely subside and usher in the chronic form of the disease, in which we have thickening of the joint, nodular formations, stiffness, incapacity, and helplessness. The blood pressure and especially the diastolic pressure is raised. The patient loses his robust appearance, muscles become flabby and emaciation and anemia supervene. The kidney function grows worse and acute exascerbations become more frequent. The blood uric acid is increased more and more. Myocardial weakness occurs, blood creatinine retention appears and the patient is gathered to his fathers.

### DIAGNOSIS.

The various arthritides have to be differentiated, especially those of gonorrhea, tuberculosis and polyarthritis deformans. The history of the case, the Von Pirket, examinations for the gonococcus, the chemistry of the blood for uric acid content, and the X rays will keep us from error. Magnus-Levy says that increased uric acid output in the urine following the administration of atophan is a diagnosis of gout.

French has demonstrated that uric acid compounds offer no resistance to X rays and in Rontgen Photograms of gouty joints the clear lines between the articulations appear unaltered, which is not true of other arthritides. If the uric acid content of the blood is still high after five days of purin free diet, gout is present according to W. His.

### PATHOLOGY.

According to Levison and Dorsett, practically all the joints of the body ultimately become affected with gout except the hip joints. There is a deposit of biurate of soda in the cartilages, cellular infiltration, thickening of the joint capsule, increase in synovia and immobility. These white chalky deposits accumulate and frequently ulcerate through the overlying skin and the patient "can write his name on a blackboard with his knuckle". The kidney becomes red and granular. The skin is frequently erythematous and eczematous. The hair is harsh and dry. The liver becomes enlarged, later fatty and cirrhosed. Myocardial degeneration occurs, the blood vascular system becomes atheromatous and the patient is carried off with apoplexy.

### TREATMENT.

A strict regimen of dietetics and hygiene should be rigidly enforced. The patient should be told frankly the success of the treatment depends entirely upon his co-operation.

Wine suppers and late hours should be absolutely prohibited. A strict avoidance of purin food, such as glandular substances like liver, kidney and brain, also peas and beans should be taken from the menu.

For convenience we advise the following diet table as used by the New York Presbyterian Hospital and the Post Graduate Medical School:

### Breakfast.

| | |
|---|---|
| Force | 10 gms. |
| Eggs | (1 egg). |
| Milk | 50 c. c. |
| Bread | 50 gms. |
| Sugar | 50 gms. |
| Butter | 30 gms. |
| Banana | 200 gms. |
| Cocoa, if desired | 50 c. c. |

### Dinner.

| | |
|---|---|
| Potato | 125 gms. |
| Eggs | (2 eggs) |
| Milk | 300 c. c. |
| Butter | 15 gms. |
| Sugar | 30 gms. |
| Apple | 300 gms. |

### Supper.

| | |
|---|---|
| Oatmeal | 10 gms. |
| Pot. cheese | 20 gms. |
| Milk | 250 c. c. |
| Bread | 100 gms. |
| Butter | 15 gms. |
| Orange | (1 or 2) |
| Water ad lib. | |

This diet yields about 70 gms. proteid and gives 2400 calories daily.

Purin days may be had on which a guarded amount of purins can be added to the purin free diet. These purin days come once or twice a week as care should be exercised to see that the patient gets enough calories.

Warm clothing should be advised and bathing and sweating is of exceeding great value. Hot fomentations to affected joints and massages are practiced in our institution. We use the galvanic current where ionization is desired. The high frequency current for its counter-irritant effect is highly beneficial. The Swedish movements are made use of. Patients are taught self-control and system. Bathing is watched carefully lest it produces undue weakness and debility. Medicinally we use the iodies and colchicum preparations, preference being given still to the wine of the leaves and to the active principal, colchicine. Atophan, a synthetic preparation is being used much in the east with very favorable results and we have had favorable results with it. We allow the drinking of our mineral waters and often a slight attack is precipitated at first due to the high sod. chloride content of the waters. It is quite common for patients who have been sent by other gout sufferers who have previously taken the cure at our place to be advised before coming to expect to get worse at the beginning "when they stay long enough to get stirred up." The doctors at Carlsbad always tell their patients they will get worse before they get better.

The gums, teeth, tonsils, gall bladder, appendix and all atri should be carefully investigated for focal infections as many arthritides are frequently both gouty and bacterial in origin.

### METHOD FOR URIC ACID OF THE BLOOD.

Pipette 5 c. c. blood into 200 c.c. casserole.

Add 25 c. c. hundredth normal acetic acid.

Boil while stirring over flame.

Add 2 c. c. alumina cream.

Boil and stir until clear.

Filter through hardened paper into second casserole, which is kept over a low flame.

Wash coagulum back into first casserole, using about 50 c. c. hot water.

Boil and filter into second casserole.

Evaporate filtrate to about 2 c. c.

Pour filtrate and wash casserole into conical centrifuge tube, keeping volume below 5 c. c.

(If cloudy, place in boiling water-bath, centrifuge, decant into dry centrifuge tube.) Cool.

Add 10 drops ammoniacal-silver-magnesium solution.

Ppts. purins aszanthin, hypozanthin.

Shake and place in cold water 15 minutes.

Centrifuge.

Pour off fluid, invert tube, let dry for 10 minutes.

Pipette 1 c. c. uric acid standard.

(1. c. c.=O. 2 mg. uris acid) into a colorimeter tube.

Add one drop of 2.5 per cent. potassium cyanide solution.

Add O.5 c. c. phosphotungstic acid solution.

Add 5 c. c. saturated sodium carbonate solution.

In 1 minute add water to 20 c. c.

To centrifuge tube add 1 or 2 drops potassium cyanide solution.

Add O. 5 c. c. phosphotungstic acid. solution.

Add 5 to 10 c. c. saturated sodium carbonate solution according to depth and color.

Transfer to second colorimeter tube.

Dilute with water to match color of standard.

The volume in the unknown tube divided by 5 mgs. uric acid in 100 c. c. blood.

PROGNOSIS AND CONCLUSIONS.

Chronic gout shortens the patient's life and makes him an easy prey for intercurrent disease. Much depends upon the co-operation of the patient. As our observation becomes clearer, and our experience broadens, and our methods of diagnosis at the Marlin Sanitarium become more efficient, we are now regarding *true* gout, as a rather rare disease.

